# Controlling the Mean Time to Extinction in Populations of Bacteria

**DOI:** 10.3390/e25050755

**Published:** 2023-05-05

**Authors:** Bhumika Thakur, Hildegard Meyer-Ortmanns

**Affiliations:** 1 School of Science, Constructor University, 28759 Bremen, Germany; 2 Complexity Science Hub Vienna, 1080 Vienna, Austria

**Keywords:** population dynamics, demographic noise, environmental fluctuations, dichotomous Markov process, normals and persisters, mean time to extinction, Gillespie simulations, WKB approach

## Abstract

Populations of ecological systems generally have demographic fluctuations due to birth and death processes. At the same time, they are exposed to changing environments. We studied populations composed of two phenotypes of bacteria and analyzed the impact that both types of fluctuations have on the mean time to extinction of the entire population if extinction is the final fate. Our results are based on Gillespie simulations and on the WKB approach applied to classical stochastic systems, here in certain limiting cases. As a function of the frequency of environmental changes, we observe a non-monotonic dependence of the mean time to extinction. Its dependencies on other system parameters are also explored. This allows the control of the mean time to extinction to be as large or as small as possible, depending on whether extinction should be avoided or is desired from the perspective of bacteria or the perspective of hosts to which the bacteria are deleterious.

## 1. Introduction

Many natural systems, such as genetic, proteomic, and cellular networks, as well as populations of bacteria on a micro-scale and populations of ecological species on a macro-scale, show demographic fluctuations due to birth and death events. At the same time, these systems are exposed to changing environments, including periodic changes, such as seasonal variations and daily changes in sunlight and darkness, and random changes, such as outbreaks of diseases, catastrophes, and climate variations. In the last decade, much activity has been invested in studying the interplay of demographic and external fluctuations and their impacts on the temporary or final fate of the respective systems [1,2,3,4,5,6,7,8,9]. Population dynamics under environmental conditions, which are frequently or even continually switching between favorable and adverse conditions, were previously studied in [1,2] in a population of two strains, one growing slightly faster than the other, particularly their fixation (one strain takes over the population) properties.

However, these studies did not consider any switching between the strains or their final fates, which we consider later. In a similar system with two strains and a randomly switching carrying capacity, the correlations between the population size and its composition were determined, particularly for conditions under which a public good produced by one of the strains is beneficial [3]. The authors of [6] focused on the establishment probability of a population and the mean time for establishment in a time-varying environment. Finally, the impact of demographic and environmental fluctuations on the mean time to extinction (MTE) was studied in a stochastic branching-annihilation process in a time-modulated environment in [7], in populations consisting of two phenotypes of bacteria in [8], and more recently, for two species interacting via competition in [9].

In more detail, the work in [8] considered a population comprising two phenotypes of bacteria, normals and persisters. Normals multiply rapidly but are less resilient to stress compared to persisters, which multiply less but are also less vulnerable. To cope with environmental stress, bacteria have developed some evolutionary strategies to survive and thrive. One such strategy is switching between their different phenotypes [10,11]. For example, it has been found that a subpopulation of persisters can survive high antibiotic concentrations [10], and the persister state is fully reversible under growth-stimulating conditions [11]. The presence of persisters considerably reduces the risk of extinction as compared to their absence. This was shown in [8] for a constant environment or a single catastrophic phase in the limiting cases of slow or fast switching. Switching is most beneficial when it is not very frequent.

Instead of a single catastrophic phase, in this paper, we consider the environmental variability to be either random (implemented as dichotomous Markov noise, characterized by the rate and amplitude) or manifest as periodic variation (either a rectangular or sine wave). The effect of unfavorable environmental conditions is simulated by reducing the birth rate of the normals. Thus, the birth rate becomes time-dependent.

We pursue two questions. From the perspective of bacteria, which want to survive as long as possible, we ask which optimal strategy of switching between phenotypes the bacteria must employ to maximize their chances of survival when the environment changes between favorable and adverse conditions at a given frequency and amplitude. On the other hand, persisters are not the only way to survive antimicrobial attacks but are at least one way to improve antibiotic resistance. In view of antibiotic resistance, we ask how to optimally tune environmental changes in terms of doses and intervals of adverse conditions so as to minimize the time to extinction of the bacterial population, given a fixed set of parameters that characterize the population of normals and persisters.

Developing tactics for successfully eradicating the microbial population and preventing antimicrobial resistance is of great relevance nowadays, and it has been the main focus of many recent studies [12]. Different types of protocols for antibiotic treatment have been discussed [13,14], particularly the so-called cycling one, which refers to the administration of drug A for T days followed by drug B for T days. In contrast, and not to be mixed up with the cycling approach, we resolve the effect of periodically varying the concentration of a single “drug” (in the form of more or less suppressed birth rates) during the total administration period for a single type of population. The focus is on the extinction of this population of bacteria beyond the mean-field limit.

Concerning the methodology, for the numerical part, we particularly used a modified Gillespie algorithm [15,16] that accounts for explicitly time-dependent propensities. As an analytical approach, we make use of the WKB method in the limit of weak perturbations or high frequencies. The extinction risk is then quantified by finding the MTE of the population.

This paper is organized as follows. In Section 2, we present the model in terms of stochastic rates with different versions of the master equations. Section 3 deals with the quasi-stationary distribution of the population, whose leakage over the course of time determines the MTE that we estimate in the subsequent sections. For a wide range of parameters and stochastically changing environments, we describe modified Gillespie simulations in Section 4. For an analytical estimate of the MTE, we derive Hamilton’s equations of motion in real and momentum space (Section 5) as they occur in the WKB approach. Section 6 deals with the WKB approach for the linear approximation of weak and periodically changing environments (Section 6.1) and the Kapitsa method for high frequencies of environmental changes (Section 6.2). Section 7 contains the discussion and a summary of the results. In the appendices, we include additional details on the numerical algorithms and the analytical approaches.

## 2. Model

Our system comprises a well-mixed two-species population considered earlier in [8]. The population consists of normals (N), whose number is denoted by *n*, and persisters (M), whose number is denoted by *m*, and their total number is *N*. Normals die at a rate set to unity throughout, and they divide at a rate B(1−n/K) per individual, where *K* is the carrying capacity for normals. The persisters neither die nor multiply as an approximation of their low birth rate in comparison to normals. Normals switch to persisters at a rate α, while persisters switch back to normals at a rate β. The ratio between the two switching rates is denoted by Γ=α/β. In summary, the birth, death, and switching events governing the population dynamics are:

(1) $$\begin{array}{ccc} N & \to & N+N\;\;\;\mathrm{at}\;a\;\mathrm{rate}\;B(1-n/K)\\ N & \to & \emptyset \;\;\;\;\;\;\;\;\;\;\mathrm{at}\;\mathrm{unit}\;\mathrm{rate}\;\\ N & \to & M\;\;\;\;\;\;\;\;\mathrm{at}\;a\;\mathrm{rate}\;\alpha \\ M & \to & N\;\;\;\;\;\;\;\;\mathrm{at}\;a\;\mathrm{rate}\;\beta \;. \end{array}$$

The stochastic nature of these events and the discreteness of the individuals result in intrinsic or demographic noise that will ultimately drive the system to extinction. All later times that we measure are therefore measured in units of the death rate. In the experiments in [10,17], the growth and switching rates are given per hour. Therefore, to understand our results in natural units, we set the death rate to be 1 death per hour, and since all other rates are in the units of the death rate, they are also in hours, without stating this explicitly.

In addition to intrinsic noise, the population is also subjected to extrinsic environmental noise. The effect of environmental perturbations is implemented by making the birth rate *B* time-dependent. These perturbations are taken to be either random (symmetric or asymmetric dichotomous Markov noise: SDMN or ADMN) or deterministic (periodic, either sinusoidal or symmetric or asymmetric rectangular).

**Random switching of the environment.** For a randomly switching environment, *B* switches between $B_{+}$ and $B_{-}$ such that $B_{-}<B_{+}$ according to ADMN, which is expressed as:

(2) $$B\left(t\right)=\frac{1}{2}\left[\left(B_{+}+B_{-}\right)+\zeta_{r}\left(t\right)\left(B_{+}-B_{-}\right)\right]$$

with the random (*r*) transition $\zeta_{r}=1\to \zeta_{r}=-1$ taking place at a rate $\nu_{+}$, while $\zeta_{r}=-1\to \zeta_{r}=1$ takes place at a rate $\nu_{-}$. The average switching rate is $\nu =(\nu_{+}+\nu_{-})/2$, and $\Delta =(\nu_{-}-\nu_{+})/2\nu$ is a measure of the switching asymmetry, with Δ=0 for the symmetric case. ADMN is stationary noise with the mean 〈ζ(t)〉=Δ and the autocorrelation function $\langle \zeta_{r}\left(t\right)\zeta_{r}\left(t^{\prime }\right)\rangle -\langle \zeta_{r}\left(t\right)\rangle \langle \zeta_{r}\left(t^{\prime }\right)\rangle =\left(1-\Delta^{2}\right)e^{-2\nu |t-t^{\prime }|}$. The stochastic system is described by a master equation, which, for a random (index r) environmental perturbation, is described by the following set:

(3) $$\begin{array}{ccc} \frac{dP^{\left(r\right)}\left(n,m,\zeta_{r},t\right)}{dt} & = & \left(E_{n}^{-}-1\right)B\left(t\right)n\left(1-\frac{n}{K}\right)P^{\left(r\right)}\left(n,m,\zeta_{r},t\right)+\left(E_{n}^{+}-1\right)nP^{\left(r\right)}\left(n,m,\zeta_{r},t\right)\\ & + & \left(E_{n}^{+}E_{m}^{-}-1\right)\alpha nP^{\left(r\right)}\left(n,m,\zeta_{r},t\right)+\left(E_{n}^{-}E_{m}^{+}-1\right)\beta mP^{\left(r\right)}\left(n,m,\zeta_{r},t\right)\\ & + & \nu_{-\zeta_{r}}P^{\left(r\right)}\left(n,m,-\zeta_{r},t\right)-\nu_{\zeta_{r}}P^{\left(r\right)}\left(n,m,\zeta_{r},t\right), \end{array}$$

where $E_{n/m}^{\pm }$ denotes shift operators such that $E_{n}^{\pm }f\left(n,m,\zeta ,t\right)=f\left(n\pm 1,m,\zeta ,t\right)$, and similarly, $E_{m}^{\pm }f\left(n,m,\zeta ,t\right)=f\left(n,m\pm 1,\zeta ,t\right)$. As indicated before, the switching rates are $\nu_{-\zeta_{r}}=\nu_{\mp }$ for $\zeta_{r}=\pm 1$ and $\nu_{\zeta_{r}}=\nu_{\pm }$ for $\zeta_{r}=\pm 1$.

**Deterministic changes in the environment.** Deterministic changes are realized as either abrupt periodic rectangular switches or continuous sinusoidal changes. For a periodic (index p) (possibly asymmetric rectangular and sinusoidal) environmental change, the birth rate varies deterministically, and the master equation reduces to

(4) $$\begin{array}{ccc} \frac{dP^{\left(p\right)}\left(n,m,t\right)}{dt} & = & \left(E_{n}^{-}-1\right)B\left(t\right)n\left(1-\frac{n}{K}\right)P^{\left(p\right)}\left(n,m,t\right)+\left(E_{n}^{+}-1\right)nP^{\left(p\right)}\left(n,m,t\right)\\ & + & \left(E_{n}^{+}E_{m}^{-}-1\right)\alpha nP^{\left(p\right)}\left(n,m,t\right)+\left(E_{n}^{-}E_{m}^{+}-1\right)\beta mP^{\left(p\right)}\left(n,m,t\right). \end{array}$$

**Abrupt periodic rectangular switches.** For a periodic rectangular wave, B(t) switches periodically between $B_{+}$ and $B_{-}$, again such that $B_{-}<B_{+}$. Again, B(t) is given by Equation (2), but with $\zeta_{r}$ replaced by $\zeta_{p}$, which enters the rectangular wave of period $T=\left(1/\nu_{+}\right)+\left(1/\nu_{-}\right)$, defined as

(5) $$\zeta_{p}\left(t\right)=\sum_{j=-\infty }^{\infty }\left[\mathrm{rect}\left(\frac{t+\frac{1}{2\nu_{+}}+jT}{1/\nu_{+}}\right)-\mathrm{rect}\left(\frac{t-\frac{1}{2\nu_{-}}+jT}{1/\nu_{-}}\right)\right],$$

where rect(.) is a rectangular function defined as rect(x)=1 if |x|<1/2 and rect(*x*) = 0 if |x|>1/2, while rect(±1/2)=0, as was also used in [2]. It becomes a square wave $\zeta_{p}\left(t\right)=\mathrm{sign}\left\{\sin\left(\pi \nu t\right)\right\}$ when the indicator of asymmetry Δ=0.

**Figure 1:**
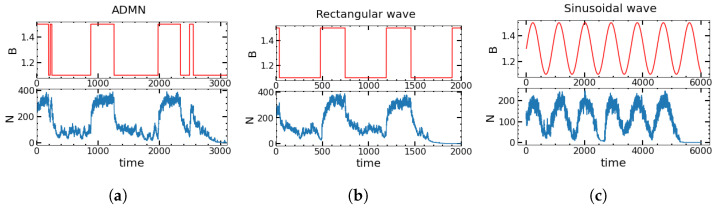
Time series of the birth rate B(t) (**upper panel**) and sample realization of the total population size *N* (**lower panel**) for (**a**) asymmetric random, (**b**) periodic rectangular, and (**c**) sinusoidal perturbations. The parameters are $B_{+}=1.5$, $B_{-}=1.1$, α=0.02, and β=0.02. For (**a**,**b**), $\left(K,\nu_{+},\nu_{-}\right)=\left(500,0.00375,0.00225\right)$, and for (**c**), $\left(K,B_{0},\omega ,\epsilon \right)=\left(300,1.3,0.007,0.2\right)$. The initial number of persisters is taken to be 0.3 K/2, and that of normals is 0.7 K/2.

**Continuous sinusoidal changes.** When B(t) changes continuously according to a periodic sinusoidal wave, it is given by $B\left(t\right)=B_{0}+\epsilon \sin\left(\omega t\right)$, where ϵ is the amplitude, and ω is the angular frequency of the perturbation. If we write the parameters of the sinusoidal perturbation in terms of the parameters of ADMN or a rectangular wave, we obtain ω=πν, $B_{0}=\left(B_{+}+B_{-}\right)/2$, and $\epsilon =(B_{+}-B_{-})/2$, but ϵ will vary independently of $B_{+}$ and $B_{-}$. Figure 1 shows an example of each type of variation in B(t) and the corresponding sample trajectories of the total population size *N* until extinction. The different modulations of the environment are visualized in Figure 1 together with the corresponding time evolution of the population size.

**Mean-field level.** Before we look further into the dynamics of these master equations, we should state what these equations yield on the mean-field level in the absence of any noise, as their fixed-point structure is needed later. In the mean-field description, the dynamics of the average number of normals $\bar{n}\left(t\right)$ and persisters $\bar{m}\left(t\right)$ are governed by the rate equations [8]

(6) $$\begin{array}{ccc} \frac{d\bar{n}}{dt} & = & B\;\bar{n}\left(1-\bar{n}/K\right)-\bar{n}-\alpha \bar{n}+\beta \bar{m}\\ \frac{d\bar{m}}{dt} & = & \alpha \bar{n}-\beta \bar{m}\;. \end{array}$$

These equations have a trivial fixed point (FP) $F_{0}$ at $\bar{n}=\bar{m}=0$, describing population extinction, and a nontrivial FP $F_{M}$ at $n_{M}=K\left(1-1/B\right)$, $m_{M}=\Gamma n_{M}$. For B>1, $F_{M}$ is stable and the population is viable, while $F_{0}$ is a saddle point. At B=1, we have a transcritical bifurcation, at which the fixed points exchange their stability properties. The relaxation time $t_{r}$ of the system in the vicinity of $F_{M}$ is determined by the principal eigenvalue of the Jacobian of the rate equation (Equation (6)), evaluated at $F_{M}$, which gives $t_{r}=2/\left(\left|-\delta -\alpha -\beta -\sqrt{-4\delta \beta +{\left(\delta +\alpha +\beta \right)}^{2}}\right|\right)∼1/\delta$, where δ=B−1, if we consider rare switching (α,β≪1) between phenotypes. 1/δ sets the time scale with which other time scales should be compared.

At this point, it should be mentioned that a detailed discussion on the mean-field level of a population of normals and persisters is provided in [18]. The rate equations are similar to Equation (6), but for exponential rather than logistic growth in the absence of medication and a different time dependence of the effective birth rate, directly reflecting the application of medication. The dynamics there were considered under the constraint of a finite overall treatment time *T* and fixed overall dosage of drugs against the bacteria. By using a multi-objective optimization approach, the best distributions of discrete medication times within the treatment duration *T* have been determined such that the numbers of surviving normals and persisters at the end of the treatment time are simultaneously minimized.

## 3. Quasi-Stationary Distribution

**Figure 2:**
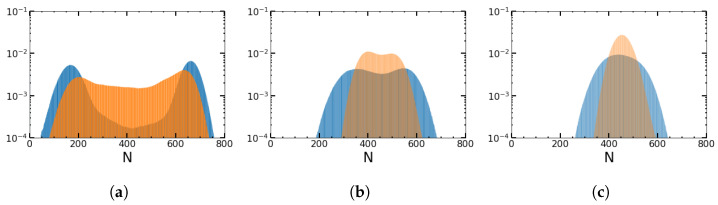
The quasi-stationary distribution for SDMN (blue color) and sinusoidal perturbations (orange color) for three values of the environmental switching frequency ν={0.001,0.1,0.5} (**a**–**c**). *N* is the sum of normals and persisters, measured from 250 sample trajectories for the distribution. Other parameters are $B_{+}=1.5$, $B_{-}=1.1$, K=1000, α=0.02, and β=0.02.

The stable FP $F_{M}$ of the deterministic system becomes metastable in the stochastic description. After a short transient of the order of $t_{r}$, the system enters the long-lived metastable state centered around $F_{M}$. This metastable state then slowly decays in time to the absorbing state n=0=m, leading to extinction. Before we determine the MTE, let us briefly describe the quasi-stationary distribution (QSD) of the population size. First, consider the case of SDMN (Δ=0, $\nu_{+}=\nu_{-}$) (blue color in Figure 2). In each of the two states corresponding to $B_{+}$ and $B_{-}$, the population tends to its metastable fixed point $F_{M_{\pm }}$ (at $n_{M_{\pm }}=K\left(1-1/B_{\pm }\right)$, $m_{M_{\pm }}=\Gamma n_{M_{\pm }}$) and fluctuates around it. As seen in previous studies as well [3,4], for low values of the frequency, the population spends more time around each of these fixed points than it spends switching between the two, and the distribution is thus bimodal (see Figure 2a). However, as the switching rate increases, the stochastic dynamics spend more time in between the fixed points (see Figure 2b). On further increasing the switching (around $\nu \approx (B_{0}-1)$), the QSD becomes unimodal, peaking at the value between the two fixed points.

However, if instead of SDMN, we have sinusoidal perturbations (orange color in Figure 2), the QSD is no longer a pronounced bimodal distribution (the remnants of a bimodal distribution may be due to the longer time that the population sees a good or adverse environment, where the slope of the sinusoidal perturbation is approximately zero) for low frequencies, as *B* continually changes and takes on all values between $B_{+}$ and $B_{-}$. Another observation is that the distribution is broader for SDMN than for the sinusoidal perturbation.

**Figure 3:**
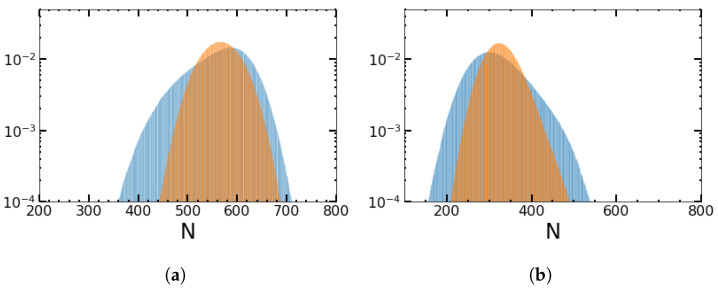
Impact of more favorable (**a**) and more adverse environmental conditions (**b**) on the quasi-stationary distribution for an ADMN (blue color) and rectangular wave perturbations (orange color): (**a**) ν=0.5, $\nu_{+}=0.25$, and $\nu_{-}=0.75$; (**b**) ν=0.5, $\nu_{+}=0.75$, and $\nu_{-}=0.25$. *N* is the sum of normals and persisters, measured from 1200 sample trajectories for the distribution. Otherwise, the conditions are the same as in Figure 2.

In Figure 3, we compare the results for ADMN and a rectangular wave with the duty cycle ($\gamma =\nu_{-}/\left(\nu_{+}+\nu_{-}\right)$) taken as (a) 0.75 and (b) 0.25, respectively. The distribution is more skewed and broader for ADMN than for the rectangular wave perturbation and shifted toward larger population sizes for higher duty cycles as in (a), in which the system sees more favorable conditions.

**Figure 4:**
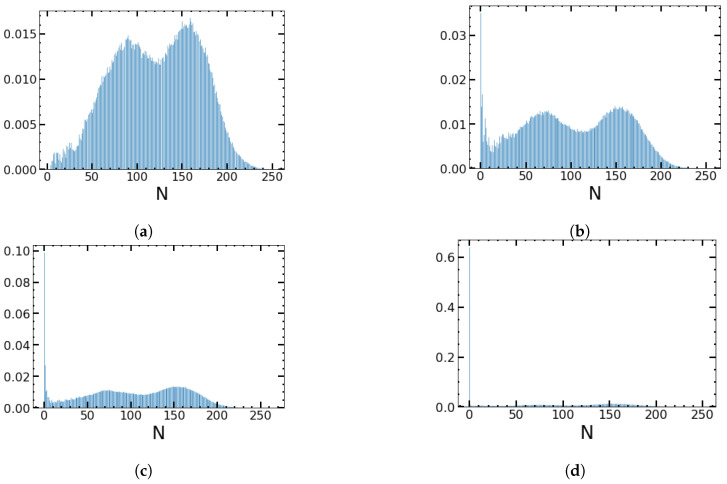
Leakage of a quasi-stationary distribution of normals and persisters, whose sum is *N*. In time windows of t∈[100–250] (**a**), t∈[350–500] (**b**), t∈[500–750] (**c**), and t∈[2300–2500] (**d**), we measure the fraction of 250 sample trajectories, which takes a value *N* as the sum of normals and persisters that have survived the respective time interval. The environment is modeled as SDMN for ν=0.05. Other parameters are $B_{+}=1.2$, $B_{-}=1.1$, K=500, α=0.02, and β=0.02.

Regardless of whether there is an environmental perturbation or not, and irrespective of the type of environmental perturbation, the QSD will eventually leak to zero on a timescale much longer than the relaxation time $t_{r}$ of the system. This is shown in Figure 4 as an example case for an SDMN perturbation. In the next sections, we discuss how long it takes on average until the distribution completely decays to zero: we determine the MTE as a function of the system parameters and the properties of the environment.

## 4. Numerical Simulations

To access a broad—and to a certain extent, complementary—parameter range as compared to analytical calculations, we directly simulated the stochastic reactions according to Equation (1). For time-independent rates of births, deaths, and phenotypic switching, but also for random switching between environmental states, we used the standard Gillespie algorithm [15]. However, for systems with time-dependent rates, such as the birth rate in our system, either a modified Gillespie algorithm or the modified next-reaction method, as discussed in [16], is at our disposal. For sinusoidal perturbations, we found it faster and easier to implement the modified Gillespie algorithm. For periodic square and rectangular waves, we used the modified next-reaction method.

We used the (modified) Gillespie algorithm in the parameter ranges α∈[0.01,0.03], β∈[0.01,0.03], and K∈[500,1000]. The reason is that the simulation time increases exponentially with large α, small β, and a large capacity *K*. The range of frequencies is determined by the saturation of the MTE to its unperturbed value. For a specific set of parameters, we then pursue the time evolution of initially N=n+m individuals of the two phenotypes until a sudden (exponentially fast) full extinction happens (caused by the instanton between the two fixed points $F_{M}$ and the extinction fixed point $F_{\emptyset }$ of Hamilton’s equations of motion). We simulated ensembles of 2400 sample trajectories to calculate the MTE for each set of parameters. The main steps of the algorithms are presented in Appendix A.1.

### 4.1. Numerical Simulations for Sinusoidal Perturbations

**Figure 5:**
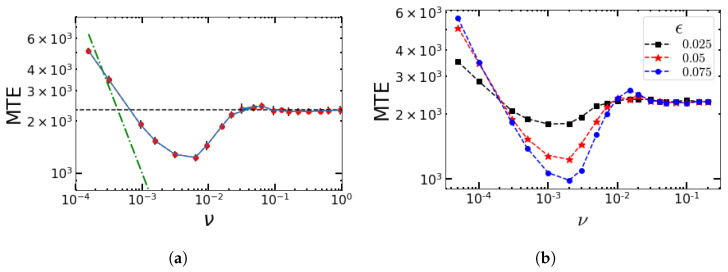
(**a**) Typical non-monotonic dependence of the MTE on the switching rate ν (or frequency ω) obtained with Gillespie simulations (red dots) for fixed α and β. The black dashed line shows the value of the MTE for the unperturbed system. The amplitude of perturbation is ϵ=0.05. The dashed green line shows the time 1/ν. For MTEs larger than that, the system on average has time to see more than one period of environmental change. (**b**) Variation in the MTE with the external frequency ν (ω) for three values of ϵ=0.025 (black squares), 0.05 (red stars), and 0.075 (blue circles). The parameters are α=β=0.02, K=500, B=1.1, and 2400 sample trajectories.

To compare the results with the random DMN and a periodic rectangular wave, we also show the simulation results for a sinusoidal perturbation with respect to the switching rate ν=ω/π. The results of the stochastic simulations show that as ν→0, the MTE reaches a maximum. As we increase ν, the MTE decreases and reaches a minimum near a certain value of ν, which is around ν=0.003 in Figure 5a. Thereafter, it again increases and approaches the unperturbed value for higher ν. For each sample trajectory, the phase of the external sinusoidal perturbation is chosen randomly from the interval (0,2π). To see how much deviation there is in the calculation of the MTE for smaller subsamples, we divided the 2400 sample trajectories for each value of ν into 12 smaller ensembles of size 200 to determine the standard deviation of the MTE calculated over the subsamples and show the results in Figure 5a (black bars) along with the MTE of all 2400 trajectories (red dots). As we shall see later, the initial time evolution that we see in Gillespie simulations will not be resolved by the linear theory in Figure 10 below.

We also illustrate the effect of the strength of perturbations on the MTE in Figure 5. As expected, the higher the value of ϵ, that is, the stronger the perturbation, the greater its effect on the MTE. The value of the minimum MTE decreases with an increase in ϵ. For an antimicrobial treatment, this has the following impact. For appropriate frequencies, the MTE is at the minimum value, and for these frequencies, the microbial population can be eliminated more quickly by increasing the dosage of antimicrobial drugs. For higher ϵ, it also takes longer for the MTE to approach the unperturbed value.

### 4.2. Impact of the Switching Rates α and β on the MTE’s Frequency Dependence for the Sinusoidal Change

**Figure 6:**
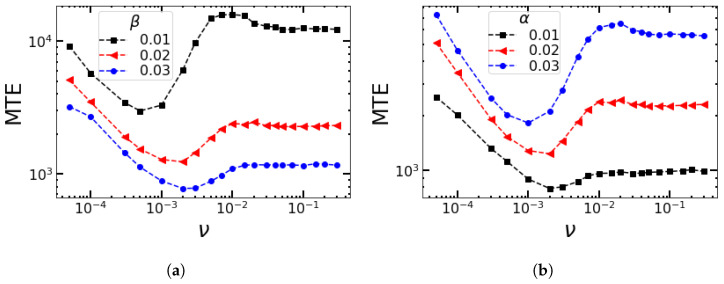
Gillespie simulations for the MTE as a function of the frequency for (**a**) different values of β and α=0.02 and (**b**) different values of α and β=0.02. Other parameters are K=500, B=1.1, ϵ=0.05, and 2400 sample trajectories.

To estimate how typical the qualitative features of Figure 5 for fixed switching parameters α and β are, we plot the MTE as a function of the frequency but with different values of α and β in Figure 6. The value of ν around which the MTE approaches its minimum value increases with an increase in β and a decrease in α.

### 4.3. Impact of the Minimum Value of the Birth Rates

As a reminder, we are mainly interested in extinction events caused by fluctuations in a quasi-stationary population, which is stable on the mean-field level and does not decay per se. Nevertheless, the choice of the minimum birth rate has some impact. The minimum birth rate can be set either above the bifurcation point of the deterministic system or below it, with the latter case chosen to be zero here. Since the impact should be more clearly visible if adverse conditions hold over a whole interval at constant $B_{-}$ (as compared to the negative phase of a sinusoidal change), to analyze the role of $B_{-}$, we chose a randomly switching environment (or—more precisely—the birth rate) between $B_{+}$ and $B_{-}$ according to SDMN (Equation (2)) and searched for remnants of the bifurcation in the deterministic system.

In all simulations (unless otherwise stated) with SDMN or a rectangular/square wave, the initial environment was chosen with the probability determined by the duty cycle γ.

**Figure 7:**
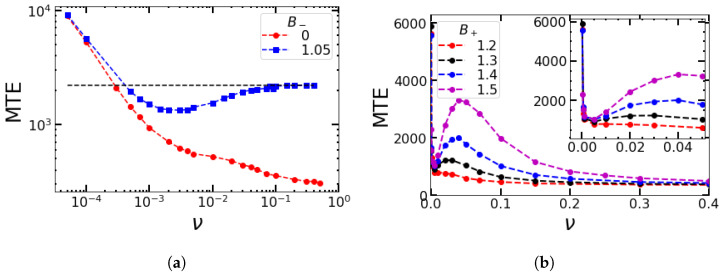
(**a**) MTE as a function of ν=ω/π when *B* switches randomly according to SDMN between $B_{+}=1.15$ and $B_{-}\in \left\{0,1.05\right\}$. (**b**) MTE as a function of ν when *B* switches randomly according to SDMN between $B_{+}=\left\{1.2,1.3,1.4,1.5\right\}$ and $B_{-}=0$. Other parameters are α=0.02, β=0.02, and K=500 in (**a**) and K=1000 in (**b**). In the inset, we zoom into ν<0.05.

**The case of $B_{-}>1$.** Let us first consider the case where *B* switches randomly between $B_{+}=1.15$ and $B_{-}=1.05$ at the same rate, that is, $\nu_{+}=\nu_{-}=\nu$. The results are shown as connected blue squares in Figure 7a.

The MTE reaches a maximum at a very low switching rate, that is, for ν→0. In the limit of low switching rates, the MTE corresponds to the average value of MTE = $(\mathrm{MTE}{|}_{B_{+}}+\mathrm{MTE}{|}_{B_{-}})/2$ for the two birth rates, while the MTE approaches ${\mathrm{MTE}|}_{(B_{+}+B_{-})/2}$ for high switching rates. The MTE reaches a minimum at around ν≈ 0.003–0.005 to increase again for more frequent switches. From ν≈0.08 onward, the MTE approaches the unperturbed value that corresponds to the averaged environment with $B_{av}=\left(B_{+}+B_{-}\right)/2=1.1$ and keeps fluctuating around this value. This frequency lies above the relaxation rate for adverse conditions ∼B−1=0.05 and below that for favorable conditions ∼B−1=0.15, so the favorable conditions are better resolved.

**The case of $B_{-}<1$.** So far, the minimum birth rate was chosen to be larger than 1, because in the deterministic limit, the fixed point $F_{M}$ loses its stability at $B=B_{-}=1$. Therefore, as soon as $B_{-}\leq 1$, the fate of the population is extinction without the need for a large stochastic fluctuation that drives it to extinction via an instanton. In the context of an antimicrobial treatment, the unfavorable environment for the microbial population would correspond to the introduction of biostatic drugs into the environment. Biostatic drugs prevent microbes from growing. When the biostatic drug only reduces the birth/growth rate of the microbes, as discussed in the case above, it is imperfect. Ideally, the biostatic drug should completely stop the growth of the microbes. Therefore, in our simulations, we also studied the case of the birth rate of normals completely going to zero under unfavorable environmental conditions, that is, $B_{-}=0$, mimicking the effect of a perfect biostatic drug.

The results are shown for $B_{+}=1.15$ and $B_{-}=0$ by red dots in Figure 7a. At low frequencies, again, the MTE reaches a maximum and is similar to the case with $B_{-}=1.05$. Then, as the switching rate increases, the MTE falls faster than it did for $B_{-}=1.05$. Under unfavorable environmental conditions, the population of the system falls exponentially, and if the duration of the unfavorable environment lasts longer than the decay time of the population, then it goes completely extinct. However, as the switching rate further increases, the conditions can improve for the population in the sense that the MTE decays less quickly around $\nu =10^{-2}$, that is, at intermediate frequencies.

This increase becomes more pronounced when we have higher birth rates in the favorable environment, as shown in Figure 7b. This is due to the fact that the environment switches more often, particularly due to favorable conditions with some finite birth rate $B_{+}>1$, which rescues the population and allows survival. Once the system sees the average birth rate $B_{av}<1$, for which it becomes less than the death rate, it corresponds to the unstable $F_{M}$ in the rate equation, and the population goes extinct very quickly with a very low value for the MTE, even before reaching the quasi-stationary distribution.

### 4.4. Deterministic versus Stochastic Changes in the Environment for Square Waves and SDMN

In general, there is a big difference for a population between being exposed to a periodically varying environment to which it can, in principle, adapt and being exposed to random changes that come as a surprise. What is the effect if the changes are almost periodically occurring, but with some fluctuations about the regular periodicity? To focus on this effect, it is natural to compare the MTEs for SDMN $\zeta_{r}\left(t\right)$ with MTEs for a square wave $\zeta_{p}\left(t\right)$, where *B* again switches between two discrete states, but regularly and periodically (see also Figure 1a). The dip in the MTE is larger for periodic switching than for random switching, but the range of ν for which the MTE is significantly lower than the unperturbed value is larger for random switching than for periodic changes (figure not displayed).

**Figure 8:**
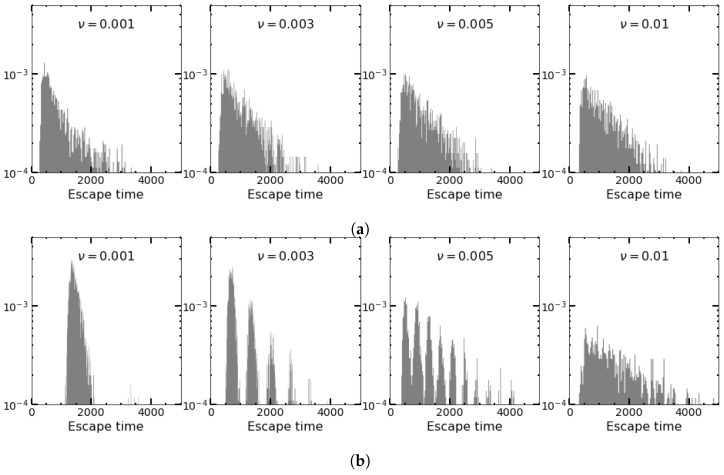
Histogram of escape times (**a**) for SDMN and (**b**) for square-wave perturbations. For square waves, all start with the zero phase, for SDMN, half start with +1 and the other half with −1. Parameters are $B_{+}=1.15$, $B_{-}1.05$, α=0.02, β=0.02, and K=500.

In Figure 8, we plot the distribution of the escape times for the symmetric random (a) and periodic (b) environments. In (a), the initial environment is chosen according to the probability decided by γ (which for the square wave is 1/2). In (b), the initial $\zeta_{p}$ is always chosen to be +1, and the phase of the square wave is taken to be zero. Therefore, in (b), all sample trajectories experience switching at the same time points. The distribution of escape times is Poissonian for random switches and shows intervals of “forbidden” extinction times for intermediate ν values in (b). The reason is that, for low ν, the system can distinguish between good and bad environmental conditions. During favorable conditions, the leakage of the quasi-stationary distribution due to fluctuations seems to be overcompensated by the resolved growing birth rate such that extinction events during these favorable conditions become very unlikely and lead to “forbidden” intervals. Note that the frequency of the environmental changes is linearly related to the number of peaks of escape times in the histogram.

As expected, for higher frequencies, as well as for random initial conditions for the deterministic changes (the latter case is not displayed), the histograms for SDMN and the square wave look similar. This means in particular that the impact of the random versus strictly periodic administration of antibiotics depends on the frequency. On the other hand, for given fixed initial conditions, there are time intervals in which an extinction is very unlikely as long as the different environmental states can be resolved.

### 4.5. Duty Cycles with Asymmetric Switching in Competition with Amplitudes

**Figure 9:**
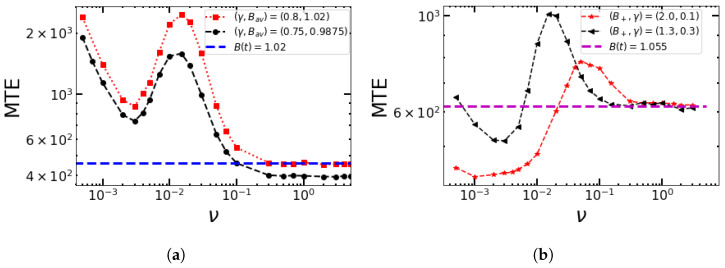
Variation in the MTE with ν for a few cases with different duty cycles γ and birth rates: (**a**) $B_{+}=1.15,\;B_{-}=0.5$; (**b**) $B_{-}=0.95,\;B_{av}=1.055$. The switching rates are $\left(\nu_{+},\nu_{-}\right)=\left(2\nu \left[1-\gamma \right],2\nu \gamma \right)$. Other parameters are α=0.02, β=0.02, and K=500.

In view of controlling the population size of bacteria, the choice of the maximum and minimum birth rates is of interest in relation to the choice of duty cycles. Can an asymmetric exposure to good or bad conditions be compensated by the more or less strong suppression of the birth rates of the population? When the individual birth rates are chosen to be the same, does it make a difference if the average birth rate $B_{av}=\gamma B_{+}+\left(1-\gamma \right)B_{-}$ is larger or smaller than 1 because of a difference in the duty cycles? The answer is given in Figure 9a with $B_{av}>1$ (red squares) and $B_{av}<1$ (black dots). The MTE for the unperturbed system with a birth rate equal to $B_{av}$ for the first case ($B_{av}>1$) is shown by the blue dashed line. $B_{av}>1$ leads to the convergence of the MTE for the unperturbed system with $B_{av}=1.02$, while $B_{av}<1$ yields short MTEs due to the unstable population, even in the deterministic limit.

In Figure 9b, we check whether a higher birth rate $B_{+}=2.0$ can compensate for a longer time spent under adverse conditions (γ=0.1) to achieve long survival. For this combination of parameters, the answer is negative. Both minimum and maximum MTEs are below the values for $B_{+}=1.3$ and γ=0.3, before the system converges to the MTE of an unperturbed system with an average birth rate of 1.055. Such observed quantitative differences in the dependencies of the MTE on the environmental switching frequency would matter in an antimicrobial treatment. From a comparison of Figure 9a,b, we conclude the following: If the effect of the antimicrobial dose lasts only for a short duration (γ>0.5 means that the bacterial population sees more favorable conditions), then in order to remove the microbial population quickly (before resistance arises), the doses should be administered at a faster rate (Figure 9a). Conversely, if the effect of the antimicrobial drug lasts for a long time (γ<0.5) (Figure 9b), the doses should be given at a lower frequency. Additionally, comparing the two curves in Figure 9b, even if $B_{+}$ is larger for the red curve, since the bad phase lasts longer, as its γ is smaller, the range of frequencies for which the MTE is smaller than the average value (magenta dashed line) is broader for the red curve.

Although our model is not supposed to advise on antibiotic treatment, we expect some qualitative features to survive more realistic modeling. While for very small frequencies, the system sees either adverse or good environmental conditions and the MTE with randomly chosen initial conditions converges to ${\mathrm{MTE}}_{l}={\left(\mathrm{MTE}\right|}_{B_{+}}+\mathrm{MTE}{|}_{B_{-}})/2$, for high frequencies, it approaches ${\mathrm{MTE}}_{h}{=\mathrm{MTE}|}_{(B_{+}+B_{-})/2}$, and due to the exponential dependence on the birth rate, ${\mathrm{MTE}}_{l}>{\mathrm{MTE}}_{h}$. Additionally, the minimum at intermediate frequencies should survive, where the frequency is low enough to resolve both environmental conditions but too high for the population to recover under good conditions. Thus, the chosen administration frequency should be smaller than the one for which the system sees the average birth rate of bacteria $\frac{B_{+}+B_{-}}{2}$ and large enough that the bacteria, independently of the starting point, see a change in environmental conditions.

## 5. WKB Approach for the MTE

In general, Gillespie results for the MTE should be complemented by analytical calculations if one wants to quantify how rare extinction events actually are. This question is of interest independently of any possible application to antibiotic treatment. In a large population, a fluctuation-induced extinction may take a rather long time. Such extinction events may not be accessible to Gillespie simulations if the simulations are too CPU–time consuming. To find the MTE to exponential accuracy, we use the Wentzel–Kramers–Brillouin (WKB) approximation for classical stochastic populations; for a review see, for example [19]. The WKB method is suitable for projecting the analysis on rare events by its very ansatz.

As it turns out, for two-phenotype populations with demographic and environmental perturbations, finding an analytical expression for the MTE becomes challenging. The analytical approaches, which we will consider below, apply to limiting cases of the parameters. For two phenotypes, these calculations partially rely on numerical approaches. We will present some analytical results for the system with periodic sinusoidal perturbations.

Let us now start with the WKB approximation. In general, there are two ways in which the WKB approximation can be applied—the real-space WKB method and the momentum-space WKB method. We summarize the results for both formulations below.

### 5.1. Real-Space WKB

We start with the WKB method in real space, as also pursued in the work of Lohmar and Meerson [8]. Let us first consider the unperturbed case by taking the birth rate *B* as a constant in the master equation (Equation (4)). As mentioned earlier, the metastable distribution slowly decays over time. At $t>>t_{r}$, $P\left(n,m,t\right)≃\pi_{n,m}\exp\left(-t/\tau \right)$ for (n,m)≠(0,0) and P(0,0,t)=1−exp(−t/τ), where τ gives the MTE and is exponentially large in *K*, the carrying capacity, while $\pi_{n,m}$ is the normalized quasi-stationary distribution.

To find the MTE, we apply the WKB-eikonal ansatz to the quasi-stationary distribution:

(7) $$\pi_{n,m}=\exp\left[-KS\left(x,y\right)\right],$$

where x=n/K and y=m/K are assumed to be continuous variables, and *K* is assumed to be sufficiently large, that is, K>>1. Using this ansatz to project on the leaking quasi-stationary solution of Equation (4) with $P^{p}\equiv P$ and expanding *S* around (x,y) to first order, we obtain a zero-energy Hamilton–Jacobi equation in the leading order of 1/K:

(8) $$H(x,y,p_{x},p_{y})=0,$$

where

(9) $$H\left(x,y,p_{x},p_{y}\right)=Bx\left(1-x\right)\left(e^{p_{x}}-1\right)+x\left(e^{-p_{x}}-1\right)+\alpha x\left(e^{-p_{x}+p_{y}}-1\right)+\beta y\left(e^{p_{x}-p_{y}}-1\right)$$

is the effective Hamiltonian, and $p_{x}=\partial S/\partial x$ and $p_{y}=\partial S/\partial y$ are the conjugate momenta. The corresponding Hamilton’s equations of motion are

(10) $$\begin{array}{ccc} \dot{x} & = & Bx\left(1-x\right)e^{p_{x}}-xe^{-p_{x}}-\alpha xe^{-p_{x}+p_{y}}+\beta ye^{p_{x}-p_{y}},\\ \dot{y} & = & \alpha xe^{-p_{x}+p_{y}}-\beta ye^{p_{x}-p_{y}},\\ \dot{p_{x}} & = & -B\left(1-2x\right)\left(e^{p_{x}}-1\right)-\left(e^{-p_{x}}-1\right)-\alpha \left(e^{-p_{x}+p_{y}}-1\right),\\ \dot{p_{y}} & = & -\beta (e^{p_{x}-p_{y}}-1)\;. \end{array}$$

The Hamiltonian *H* does not explicitly depend on time, and therefore, H=E=0 is an integral of motion. If *H* is time-dependent, such as for a time-dependent birth rate B(t), this is no longer true. There are three zero-energy saddles FP of the Hamiltonian flow: (0,0,0,0), (1−1/B,Γ(1−1/B),0,0), and (0,0,−lnB,−lnB). The first two correspond to $F_{0}$ and $F_{M}$, respectively, and the third saddle is called a fluctuational extinction point, which is denoted by $F_{\emptyset }$. The instanton is a heteroclinic trajectory starting at the metastable FP $F_{M}$ at time −∞ and approaching the extinction fixed point FP $F_{\emptyset }$ at time t=+∞. To find the MTE, we need to determine the action along this heteroclinic trajectory:

(11) $$S=\int (p_{x}dx+p_{y}dy-Hdt)\;.$$

Since the Hamiltonian is nonintegrable, we obtain the instanton numerically using an iterative numerical scheme, detailed in Appendix A.2. From the action, the MTE τ can be obtained with exponential accuracy from τ∼exp(KS).

### 5.2. Momentum-Space WKB

For comparison and later use, we next outline the WKB method in momentum space. This method involves deriving a linear partial differential equation (PDE) for the probability-generating function. We define the probability-generating function *G* as

(12) $$G\left(\rho_{1},\rho_{2},t\right)=\sum_{n,m=0}^{\infty }\rho_{1}^{n}\rho_{2}^{m}P\left(n,m,t\right),$$

where $\rho_{1}$ and $\rho_{2}$ are auxiliary variables. Once $G(\rho_{1},\rho_{2},t)$ is found, the probabilities P(n,m,t) are given by the coefficients of its Taylor expansion around $\rho_{1}=\rho_{2}=0$. Multiplying both sides of the master equation (Equation (4)) by $\rho_{1}^{n}\rho_{2}^{m}$ and summing over *n* and *m* gives an evolution equation for the generating function that, in our case, reads:

(13) $$\begin{array}{ccc} \frac{\partial G}{\partial t}= & B & \rho_{1}^{2}\frac{\partial G}{\partial \rho_{1}}-\frac{B}{K}\left(\rho_{1}^{2}\frac{\partial G}{\partial \rho_{1}}+\rho_{1}^{3}\frac{\partial^{2}G}{\partial \rho_{1}^{2}}\right)-B\rho_{1}\frac{\partial G}{\partial \rho_{1}}+\frac{B}{K}\left(\rho_{1}\frac{\partial G}{\partial \rho_{1}}+\rho_{1}^{2}\frac{\partial^{2}G}{\partial \rho_{1}^{2}}\right)\\ & + & \left(1-\rho_{1}\right)\frac{\partial G}{\partial \rho_{1}}+\left(\rho_{2}-\rho_{1}\right)\left(\alpha \frac{\partial G}{\partial \rho_{1}}-\beta \frac{\partial G}{\partial \rho_{2}}\right). \end{array}$$

Making use of the eikonal ansatz $G\left(\rho_{1},\rho_{2},t\right)=\exp\left[-S^{\prime }\left(\rho_{1},\rho_{2},t\right)\right]$ with $S^{\prime }$ as the action in momentum space in Equation (13) and neglecting $\partial^{2}S^{\prime }/\partial \rho_{\left\{1,2\right\}}^{2}$, we obtain the following Hamilton-Jacobi equation that defines $H^{\prime }$:

(14) $$H^{\prime }=0=\frac{\partial S^{\prime }}{\partial t}+\left(\rho_{1}-1\right)\left[\left(B\left(1-1/K\right)\rho_{1}-1\right)Q_{1}-B/K\rho_{1}^{2}Q_{1}^{2}+\left(\alpha Q_{1}-\beta Q_{2}\right)\left(\rho_{2}-\rho_{1}\right)\right],$$

where $Q_{1}=-\partial S^{\prime }/\partial \rho_{1}$ and $Q_{2}=-\partial S^{\prime }/\partial \rho_{2}$ are the canonically conjugate coordinates to the momenta $\rho_{1},\rho_{2}$. Shifting the momenta $p_{1}=\rho_{1}-1$, $p_{2}=\rho_{2}-1$ and taking $Q_{1}=q_{1}K$, $Q_{2}=q_{2}K$, B(1−1/K)≈B, assuming K>>1, gives the Hamiltonian

(15) $$H=H^{\prime }/K=\left(p_{1}q_{1}\left[\left(-1+B(1+p_{1})\right)-Bq_{1}{\left(1+p_{1}\right)}^{2}\right]+\left(-p_{1}+p_{2}\right)\left(q_{1}\alpha -q_{2}\beta \right)\right),$$

and the action $S\equiv S^{\prime }/K$. Hamilton’s equations of motion in momentum space, which we will use later, are then given by

(16) $$\begin{array}{ccc} \dot{q_{1}} & = & q_{1}\left[-1+B\left(1+2p_{1}-\left(1+p_{1}\right)\left(1+3p_{1}\right)q_{1}\right)\right]+\beta q_{2}-\alpha q_{1}\\ \dot{q_{2}} & = & \alpha q_{1}-\beta q_{2}\\ \dot{p_{1}} & = & -p_{1}\left(-1+B(1+p_{1})\right)+2Bp_{1}{\left(1+p_{1}\right)}^{2}q_{1}-\alpha \left(p_{2}-p_{1}\right)\\ \dot{p_{2}} & = & \beta (p_{2}-p_{1})\;. \end{array}$$

The fixed points become (0,0,0,0), $\left(\frac{-1+B}{B},\frac{-1+B}{B}\Gamma ,0,0\right)$, and $\left(0,0,\frac{1-B}{B},\frac{1-B}{B}\right)$. Note that we can go from the momentum-space Hamiltonian to the real-space Hamiltonian by using the transformation $p_{1}\to e^{p_{x}}-1$, $p_{2}\to e^{p_{y}}-1$, $q_{1}\to xe^{-p_{x}}$, and $q_{2}\to ye^{-p_{y}}$ or, more generally, by using the transformation $p_{x_{m}}\to e^{p_{x_{r}}}-1$, $p_{y_{m}}\to e^{p_{y_{r}}}-1$, $x_{m}\to x_{r}e^{-p_{x_{r}}}$, and $y_{m}\to y_{r}e^{-p_{y_{r}}}$, where $\left\{x_{r},y_{r}\right\}$ and $\left\{p_{x_{r}},p_{y_{r}}\right\}$ are coordinates and momenta in real space, and $\left\{x_{m},y_{m}\right\}$ and $\left\{p_{x_{m}},p_{y_{m}}\right\}$ are coordinates and momenta in momentum space. From here onward, we use the momentum-space Hamiltonian, as it is more convenient to work with when we discuss the Kapitsa method for high frequencies.

Before we look at the effects of environmental perturbations on the MTE of the population in the next section, let us first briefly discuss the unperturbed case. It was shown in [8] that, close to the bifurcation point B=1, the separation of timescales for the dynamics of slow variables (persisters) and fast variables (normals) can be used to obtain the expression for the MTE in a constant environment, which is given by $\tau ≃\exp\left[K\delta^{2}\left(1/2+\Gamma \right)\right]$. Since Γ=α/β, this means that the MTE increases exponentially with the carrying capacity *K* and the switching rate from normals to persisters α, but the inverse is true for the switching rate from persisters to normals $\beta^{-1}$. This dependence also holds outside the validity range for which it was derived in [8]. Due to the exponential dependence of the MTE on the system parameters, the stochastic simulations become computationally expensive as we increase α and *K* or decrease β. This acts as a practical constraint on the range of parameters that we can explore in our simulations.

## 6. Effects of a Changing Environment: Analytical Approaches

We study the effect of an environmental perturbation by making the birth rate time-dependent. We consider the periodic sinusoidal perturbation in two limits: the linear approximation for small-amplitude perturbations and the Kapitsa approximation for high-frequency perturbations.

### 6.1. Sinusoidal Changes in the Environment for Weak Perturbations

We consider the environmental perturbation to vary sinusoidally in time with a frequency ω and an amplitude ϵ such that

(17) $$B\left(t\right)=B_{0}+\epsilon \sin\omega t.$$

The Hamiltonian in momentum space is now time-dependent:

(18) $$H\left(q_{1},q_{2},p_{1},p_{2},t\right)=H_{0}\left(q_{1},q_{2},p_{1},p_{2}\right)+\epsilon H_{p}\left(q_{1},q_{2},p_{1},p_{2},t\right),$$

where $H_{0}$ is the unperturbed Hamiltonian given by Equation (15), 0≤ϵ≤1, while

(19) $$H_{p}=-q_{1}p_{1}\left(1+p_{1}\right)\left(-1+q_{1}+q_{1}p_{1}\right)\sin\omega t$$

is the time-dependent Hamiltonian resulting from the environmental perturbations.

In the limit of ϵ<<1, the action along the extinction trajectory is $S\left(t_{0}\right)=S_{0}+\Delta S\left(t_{0}\right)$, where $t_{0}$ is a yet-to-be-determined phase (the optimal time instant to escape), and $S_{0}$ is the action along the unperturbed path, while ΔS is the correction to the action [7]. Assuming that the perturbed Hamiltonian $\epsilon H_{p}$ is too small to affect the extinction trajectory obtained from the unperturbed Hamiltonian, the minimum action barrier along the same trajectory can be calculated as

(20) $$\Delta S=\min_{t_{0}}\{-\epsilon \int_{-\infty }^{\infty }H_{p}\left(t,t_{0}\right)dt\}.$$

The minimum additional action can thus be numerically obtained by varying the phase difference $t_{0}\in \left(0,T\right]$, where T=2π/ω. The MTE is then estimated as $\tau \propto \exp K(S_{0}+\Delta S)$.

**Figure 10:**
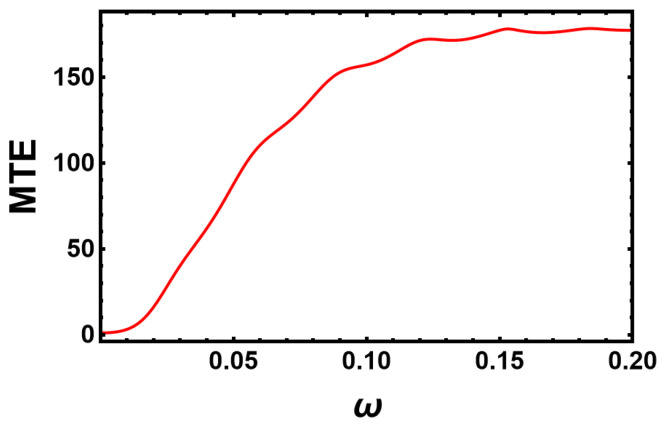
Variation in the MTE as a function of the frequency of the perturbation predicted by the linear theory for K=500, α=β=0.02, B=1.1, and ϵ=0.05. The maximum reduction in the MTE due to the perturbation in the birth rate is observed at small frequencies. For high frequencies (ω>>δ), the system sees an average of the environmental perturbation, which equals the unperturbed case, and thus, the MTE approaches the unperturbed value.

In Figure 10, we show the variation in the MTE as a function of ω. The unperturbed action $S_{0}$ is calculated along the extinction trajectory using the iterative scheme detailed in Appendix A.2. The correction to the action ΔS is then calculated along the same trajectory using Equation (20). According to Figure 10, the linear theory predicts that the MTE reaches a minimum for ω→0. As ω increases, the MTE also increases and eventually approaches the unperturbed value for ω>δ (with δ approximately the inverse relaxation time). The wiggling form of the curve seems to be a numerical artifact. The linear theory does not give correct predictions below ν (or corresponding ω)=0.003. Beyond this value, it predicts that the MTE increases or the correction to the action decreases with a further increase in ν (or ω). Eventually, it approaches its unperturbed value for higher frequencies.

A quantitative comparison between results for the MTE from the linear theory and Gillespie simulations shows a clear discrepancy. This may be due to different reasons. First of all, the WKB approach gives the MTE only up to a pre-exponential factor that we have not determined, as Gillespie simulations for large population sizes become very time-consuming. Moreover, the pre-exponent may also depend on ω. However, the prefactor does not explain the order-of-magnitude difference. Errors also arise due to the action calculated using an iterative scheme that involves a discretization error and a cut-off of the integration time. The error in the iterative scheme is exaggerated due to the exponential dependence of the MTE on S, which is also multiplied by K, which exaggerates it even more. This explains why the MTEs in Figure 5 differ by an order of magnitude from those in Figure 10 when they fluctuate about the unperturbed value at high frequencies and are calculated either analytically or numerically.

**Figure 11:**
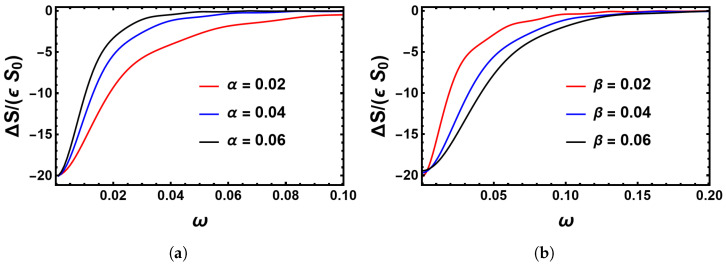
(**a**) Correction to the action ΔS, rescaled by the unperturbed action $S_{0}$ for three values of α, while β=0.02. As α increases, the correction to the action approaches zero more rapidly; that is, the range of frequencies for which the environmental perturbation has a significant effect on the system decreases as α increases. (**b**) The same as in (**a**), but for three values of β for α=0.02. Here, as β increases, the range of frequencies for which the environmental perturbation has a significant effect on the system increases together with β.

In Figure 11a,b, we compare the correction to the action ΔS, rescaled by ϵ times the unperturbed action $S_{0}$, as predicted by the linear theory for three values of α and β, respectively. These results show that the range of ω, for which the correction to the action ΔS is significant, decreases as we increase α or as we decrease β. This tells us that the MTE approaches its unperturbed value at a lower value of ω as we increase α, whereas, as we increase β, the corresponding value of ω increases. Opposing behaviors between the α and β dependence were also seen in the stochastic simulations presented in Figure 6, but there it was for the shift of the location of the minimum MTE.

**Figure 12:**
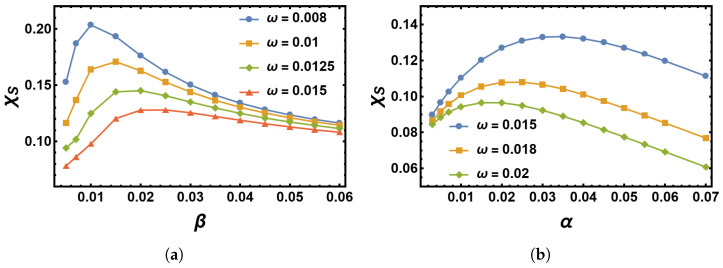
(**a**) Logarithmic susceptibility $\chi_{S}$ as a function of β for four values of ω with a single peak in $\chi_{S}$ for α=0.02. (**b**) The same as (**a**), but as a function of α for three values of ω with a single peak in $\chi_{S}$ for β=0.02.

It should be noticed that the correction ΔS/ϵ with ΔS of Equation (20) may be interpreted as the linear response of the MTE $\propto \exp K(S_{0}+\Delta S)$ to an external perturbation δh≡ϵsinωt. The response is then evaluated at the best time instant $t_{0}$ (that is, the instant with the smallest entropic barrier) to escape along the optimal path that minimizes the escape barrier, where the optimal path is still assumed to be the unperturbed one. Therefore,

(21) $$|\lim_{\delta h\to 0}\Delta S/\epsilon |=\min_{t_{0}}\{-\int_{-\infty }^{\infty }H_{p}\left(t,t_{0}\right)dt\}=\left|\lim_{\delta h\to 0}\frac{\delta \log\mathrm{MTE}}{\delta h}\right|\equiv \chi_{S}$$

has the interpretation of a logarithmic susceptibility $\chi_{S}$ (the index *S* is reminiscent of the action *S*), as is also considered in [7,9,20]. Here, this quantity indicates the sensitivity of the MTE to the system parameters *B*, β, and α for given frequencies ω. We plot this dependence of β and α in Figure 12a,b, respectively. The variation is non-monotonic in both cases and peaks at certain values of β and α, respectively, which increases (decreases) with an increase in ω of the environmental switching. Non-monotonicity was also observed in [9]. This means that at certain switching rates α and β, the system is most susceptible to perturbations.

### 6.2. Sinusoidal Changes of the Environment for High Frequencies

**Figure 13:**
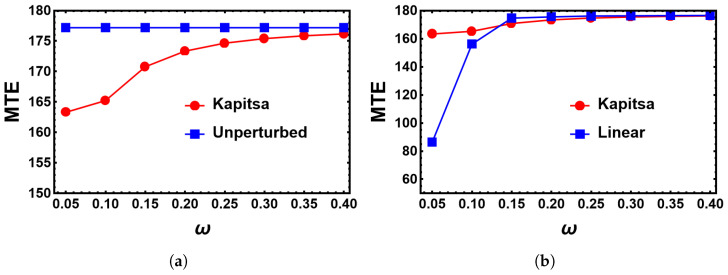
MTE as obtained from the Kapitsa approximation (red dots) as a function of the frequency in comparison to (**a**) the unperturbed case (blue squares) and (**b**) the linear approximation (blue squares). Parameters are K=500, α=β=0.02, B=1.1, and ϵ=0.05.

Another limit that is analytically approximately accessible is the limit of high frequencies ω>>δ. Here, we calculate the correction to the action, which turns out to be of the second order in ϵ, by using the Kapitsa method [7]. These corrections are small even if ϵ is of the order of 1. Here, we only summarize the main steps. The details are included in the appendix. Naturally, one expects that for a fast-changing environment, the system sees only a kind of time-independent average. Thus, the goal is to derive a time-independent effective Hamiltonian, splitting the coordinates into slow ($X_{i},Y_{i}$) and fast ($\xi_{i},\eta_{i}$) variables, i∈{1,2}. The first step is to derive Hamilton’s equations of motion for the fast variables, neglecting terms of the second order in $\xi_{i},\eta_{i}$. These equations can be integrated over time and lead to the relations $\xi_{i}=\xi_{i}\left(X_{i},Y_{i},t\right)$ and $\eta_{i}=\eta_{i}\left(X_{i},Y_{i},t\right)$. The Hamiltonian in the new slow variables should be equivalent to the one in the original variables; thus, the transformation from old to new coordinates and momenta should be canonical and satisfy Poisson brackets. As it turns out, when choosing the transformation from $\left(q_{i},p_{i},t\right),i\in \left\{1,2\right\}$ according to $q_{i}\left(t\right)=X_{i}\left(t\right)+\xi_{i}\left(t\right)$, $p_{i}\left(t\right)=Y_{i}\left(t\right)+\eta_{i}\left(t\right)$ by replacing $\xi_{i}=\xi_{i}\left(X_{i},Y_{i},t\right)$, $\eta_{i}=\eta_{i}\left(X_{i},Y_{i},t\right)$ accordingly, the Poisson brackets vanish only up to $O{\left(\epsilon /\omega \right)}^{2}$. In order to obtain the first non-vanishing correction to the Hamiltonian, which is itself already of the order of $\epsilon^{2}$, we use the so-obtained relations between old and new variables only as a starting point to guess a transformation that satisfies the Poisson brackets up to $O{\left(\epsilon /\omega \right)}^{3}$. The generating function $F_{2}\left(q_{i},Y_{i},t\right)$ of this transformation determines the new Hamiltonian according to $H^{\prime }\equiv H+\partial F_{2}/\partial t$, which is then averaged over one time period of rapid oscillations to result in the desired time-independent Hamiltonian. Its equations of motion are solved by the iteration method in Appendix A.2. The results are shown in Figure 13. The MTEs of the Kapitsa method approach the unperturbed values as they should for high frequencies (Figure 13a) and also those of the linear approximation for large frequencies, while the Kapitsa method is not reliable for low frequencies.

## 7. Discussion and Summary of the Results

We considered populations of normals and persisters under deterministically and stochastically varying environments. Both versions were realized under symmetric or asymmetric durations of the periods of favorable and adverse environmental conditions, characterized by the amplitude and frequency of the perturbation. An answer to our first question of how the bacteria should best adapt to a given changing environment is to choose the switching rate of normals to persisters to be as large as possible and that from persisters back to normals to be as small as possible, as the MTE increases (decreases) exponentially with increasing α (decreasing β), respectively. Nevertheless, we observed a non-monotonic *change* in the exponential growth or decay that became visible in the logarithmic susceptibility with respect to the external perturbation. For an increasing frequency of environmental changes, the maximum sensitivity shifted to smaller (larger) values of α (β), respectively.

More pronounced is the non-monotonic dependence of the MTE when it is plotted as a function of the frequency of the environmental change between favorable and adverse conditions. Here, only the Gillespie simulations could resolve the limit of rather low frequencies. The MTE is largest for very low frequencies close to zero and initially decreases with an increase in the frequency, rapidly dropping to a minimum value that lies below the MTE for the unperturbed population. Upon further increasing the frequency, the MTE increases again by possibly overshooting the unperturbed MTE value. The unperturbed value is finally approached for high frequencies, as the system then sees only an average about the unperturbed value over the environmental conditions if the fluctuations have symmetric amplitudes about the unperturbed birth rate.

Thus, the answer to our second question of how to tune the amplitude and frequency of applying adverse conditions to the bacteria to eliminate the population is just provided by this non-monotonic behavior: choosing the dose of applications (amplitude in our model) to be as large as possible (and still tolerable for the host of the bacterial population) and a frequency outside of the small interval, in which the MTE is minimum. Adverse conditions chosen so as to tune the minimum birth rate to zero or at least below the bifurcation, from which on the population is unstable in the deterministic limit, turned out to be advantageous, as they impede the later resurrection of the population under favorable conditions.

Further, let us compare the impact of symmetric versus asymmetric durations of favorable and adverse conditions. Here, the MTE is continuously reduced with an increase in the fraction of time that the population is exposed to adverse conditions (keeping all other parameters fixed). A qualitative change in the dependence of the MTE on the frequency is observed when adverse conditions push the birth rate below the bifurcation threshold (in the deterministic limit): before any quasi-stationary population distribution is established, it already goes extinct.

As to the comparison between deterministic and stochastic changes in the environment, one may have expected a mere broadening of both the quasi-stationary distributions and the spectrum of the times to extinction, that is, the probability distribution of extinction times. However, the distribution of extinction times looks qualitatively different for rectangular waves with duty cycles and ADMN of the same average rates $\nu_{+},\nu_{-}$ and amplitudes $B_{+},B_{-}$. For ADMN, we measured a histogram that is fitted by a Poissonian. For the rectangular wave, we found a histogram in which entire intervals of extinction times seem to be forbidden for the sample trajectories of the population when always starting from the same initial conditions. This may reflect the fact that a population will most likely not go extinct in a certain time interval as long as the environmental conditions are resolved by the population to become increasingly favorable so that these conditions overcompensate for the inherent leakage of the quasi-stationary distribution.

In view of further applications to microbial populations of bacteria, a natural extension of this work would be the inclusion of competition between normals and persisters. Maintaining part of the population as persisters (not reproductive and being beneficial only “in case”) amounts to insurance for the population or a luxury that has its price. This price may be paid by including competitive interactions between the two phenotypes. Another extension would be the inclusion of irreversible mutations in response to environmental variation leading to resistance rather than persistence that we considered so far. 

## Data Availability

Not applicable.

## Appendix A. Numerical Methods

### Appendix A.1. Stochastic Simulations

As mentioned in the main text, we used the standard Gillespie algorithm [15] for time-independent (birth) rates and either the modified Gillespie algorithm or the modified next-reaction method by Anderson [16] for time-dependent rates. For details on the modified next-reaction method, we refer to [16]. In the following, we summarize the main steps of both algorithms.

#### Appendix A.1.1. Gillespie Algorithm

##### Time-Independent Rates

1. Initialize the algorithm by setting the initial number of normals (n) and persisters (m) and setting t=0.
2. Calculate the propensity function for each reaction. In the absence of any environmental perturbation, we have four stochastic reactions i∈{1,...,4} (birth of normals, death of normals, switching from normals to persisters, and switching from persisters to normals), each with a propensity function $a_{i}\in \left\{Bn\left(1-n/K\right),n,\alpha n,\beta m\right\}$, respectively. In the presence of ADMN, there are two additional stochastic reactions corresponding to environmental switching $\xi_{r}\to -\xi_{r}$.
3. Set $a_{0}=\sum_{i=1}^{M}a_{i}$, where M=4 for the unperturbed system and M=6 for ADMN.
4. Generate two random numbers $r_{1}$ and $r_{2}$ from a uniform distribution U(0,1).
5. Find the time until the next reaction should take place, that is, $\Delta t=1/a_{0}\ln\left(1/r_{1}\right)$.
6. Find the reaction μ∈[1,…,M] that takes place such that
  $$\sum_{i=1}^{\mu -1}a_{i}<r_{2}a_{0}\leq \sum_{i=1}^{\mu }a_{i}.$$
7. Set t=t+Δt and update the number of normals (n) and persisters (m) according to the reaction μ.
8. Return to step 2 or quit.

##### Time-Dependent Rates: Modified Gillespie Algorithm

When we have time-dependent propensities, that is, when *B* varies periodically with time, the time until the next reaction is found by solving the following equation for Δt:

(A1) $$\sum_{i=1}^{4}\int_{t}^{t+\Delta t}a_{i}\left(n\left(t\right),m\left(t\right),s\right)ds=\ln\left(1/r_{1}\right).$$

The reaction μ that fires at that time is chosen according to the probabilities $a_{i}\left(n\left(t\right),m\left(t\right),t+\Delta t\right)/a_{0}$ according to step 6, where $a_{0}=\sum_{i=1}^{M}a_{i}\left(n\left(t\right),m\left(t\right),t+\Delta t\right)$ [16].

#### Appendix A.1.2. Modified Next-Reaction Method

The modified next-reaction method from [16] and the next-reaction method from [21], on which it is based, make use of the fact that the reaction times of the systems can be represented as the firing times of Poisson processes with internal times given by integrated propensity functions. Let the internal time for the *i*th reaction or process be $T_{i}=\int_{0}^{t}a_{i}\left(n\left(s\right),m\left(s\right),s\right)ds$ and let $P_{i}$ be the first firing time of the *i*th process. We define $\Delta t_{i}$ as the amount of time that must pass in order for the *i*th reaction to fire. The main steps of the algorithm are:

1. Initialize the algorithm by setting the initial number of normals (n) and persisters (m) and setting t=0. For each *i*, set $T_{i}=0$.
2. Generate *M* random numbers $r_{i}$ from a uniform distribution $r_{i}\in U\left(0,1\right)$ and set $P_{i}=\ln\left(1/r_{i}\right)$ for each *i*.
3. Calculate $\Delta t_{i}$ by solving $\int_{t}^{t+\Delta t_{i}}a_{i}\left(n\left(t\right),m\left(t\right),s\right)ds=P_{i}-T_{i}$ for $\Delta t_{i}$.
4. Set $\Delta t=\min_{i}\left\{\Delta t_{i}\right\}$ and let $\Delta t_{\mu }$ be the time for which the minimum is realized, that is, let $\Delta t_{i}$ be the minimum for the reaction i=μ.
5. Increase the time by an increment of t=t+Δt and update the number of normals and persisters according to the reaction μ.
6. For each *i*, set $T_{i}=T_{i}+\int_{t}^{t+\Delta t_{i}}a_{i}\left(n\left(t\right),m\left(t\right),s\right)ds$.
7. For the reaction μ, let *r* be a uniform random number r∈U(0,1), and set $P_{\mu }=P_{\mu }+\ln\left(1/r\right)$.
8. Return to step 3 or quit.

For time-independent rates and environmental switching between discrete states (ADMN and periodic rectangular wave), the algorithm becomes simpler. For time- independent rates and ADMN (see Algorithm 3 in [16]), the equation to find $\Delta t_{i}$ simplifies to $\Delta t_{i}=\left(P_{i}-T_{i}\right)/a_{i}$, and the update to $T_{i}$ simplifies to $T_{i}=T_{i}+a_{i}\Delta t$. In the case of a periodic rectangular wave, the propensity function for the birth of normals becomes $a_{1}=B\left(\xi_{p}\left(t\right)\right)n\left(1-n/K\right)$. Since *B* is discrete and therefore a constant in each iteration, the integral in step 3 can be solved easily to find $\Delta t_{i}$, from which Δt is determined in step 4. If $\Delta t>\Delta t_{switch}$, where $\Delta t_{switch}$ is the time to the next switch in the environment, we switch ξ→−ξ and propagate the time to $t\to \Delta t_{switch}$. See the supplemental material to [2] for additional explanations of the algorithm for a periodic rectangular wave.

### Appendix A.2. The Adapted Chernykh-Stepanov Iteration Method

For a numerical solution of Hamilton’s equations of motion to find the extinction trajectory, we used the Chernykh-Stepanov iteration method [22]. More precisely, we used an adaptive version of the algorithm, as used in [8]. In this scheme, the starting iteration numerically integrates the Hamilton’s equations for coordinates forward in time by fixing the momenta at their final values (here, at the extinction fixed point). The resulting coordinate trajectory is then used to fix the coordinates in the Hamilton’s equations for momenta, which are then integrated backward in time, starting from some time $t=t_{max}$ going down to t=0. This back-and-forth iteration method is repeated until the path converges to the desired instanton solution.

## Appendix B. The Kapitsa Method for High Frequencies

We consider the high-frequency limit (ω≫1) and calculate the small high-frequency corrections to the unperturbed coordinates and momenta of the system. Separating slow and fast timescales leads to:

(A2) $$\begin{array}{c} q_{1}\left(t\right)=X_{1}\left(t\right)+\xi_{1}\left(t\right)\;\;q_{2}\left(t\right)=X_{2}\left(t\right)+\xi_{2}\left(t\right)\\ p_{1}\left(t\right)=Y_{1}\left(t\right)+\eta_{1}\left(t\right)\;\;p_{2}\left(t\right)=Y_{2}\left(t\right)+\eta_{2}\left(t\right), \end{array}$$

where $X_{1}$, $X_{2}$, $Y_{1}$, and $Y_{2}$ are slow variables, and $\xi_{1}$, $\xi_{2}$, $\eta_{1}$, and $\eta_{2}$ are rapidly oscillating variables. Expanding the Hamiltonian $H(q_{1},q_{2},p_{1},p_{2},t)$ around $q_{1}=X_{1}$, $q_{2}=X_{2}$, $p_{1}=Y_{1}$, and $p_{2}=Y_{2}$ up to the second order in $\xi_{1}$, $\xi_{2}$, $\eta_{1}$, and $\eta_{2}$, we obtain:

(A3) $$\begin{array}{ccc} H(q_{1},q_{2},p_{1},p_{2},t) & = & H\left(X_{1},X_{2},Y_{1},Y_{2}\right)+\xi_{1}\frac{\partial H}{\partial X_{1}}+\xi_{2}\frac{\partial H}{\partial X_{2}}+\eta_{1}\frac{\partial H}{\partial Y_{1}}+\eta_{2}\frac{\partial H}{\partial Y_{2}}\\ & + & \frac{1}{2}\xi_{1}^{2}\frac{\partial^{2}H}{\partial X_{1}^{2}}+\frac{1}{2}\xi_{2}^{2}\frac{\partial^{2}H}{\partial X_{2}^{2}}+\frac{1}{2}\eta_{1}^{2}\frac{\partial^{2}H}{\partial Y_{1}^{2}}+\frac{1}{2}\eta_{2}^{2}\frac{\partial^{2}H}{\partial Y_{2}^{2}}+\xi_{1}\xi_{2}\frac{\partial^{2}H}{\partial X_{1}\partial X_{2}}\\ & + & \xi_{1}\eta_{1}\frac{\partial^{2}H}{\partial X_{1}\partial Y_{1}}+\xi_{1}\eta_{2}\frac{\partial^{2}H}{\partial X_{1}\partial Y_{2}}+\xi_{2}\eta_{1}\frac{\partial^{2}H}{\partial X_{2}\partial Y_{1}}+\xi_{2}\eta_{2}\frac{\partial^{2}H}{\partial X_{2}\partial Y_{2}}\\ & + & \eta_{1}\eta_{2}\frac{\partial^{2}H}{\partial Y_{1}\partial Y_{2}}\equiv \tilde{H} \end{array}$$

with $\tilde{H}$ being the truncated Hamiltonian in terms of slow and fast variables. The Hamilton’s equations become

(A4) $$\begin{array}{ccc} \dot{q_{1}} & = & \dot{X_{1}}+\dot{\xi_{1}}\approx \frac{\partial \tilde{H}}{\partial Y_{1}}\;\;\;\dot{q_{2}}=\dot{X_{2}}+\dot{\xi_{2}}\approx \frac{\partial \tilde{H}}{\partial Y_{2}}\\ \dot{p_{1}} & = & \dot{Y_{1}}+\dot{\eta_{1}}\approx -\frac{\partial \tilde{H}}{\partial X_{1}}\;\dot{p_{2}}=\dot{Y_{2}}+\dot{\eta_{2}}\approx -\frac{\partial \tilde{H}}{\partial X_{2}}. \end{array}$$

Demanding that the rapidly oscillating terms in Equation (A4) balance each other gives

(A5) $$\begin{array}{c} \dot{\xi_{1}}=-\epsilon X_{1}\left[-1-2Y_{1}+X_{1}\left(1+Y_{1}\right)\left(1+3Y_{1}\right)\right]\sin\omega t\\ \dot{\xi_{2}}=0\\ \dot{\eta_{1}}=\epsilon Y_{1}\left(1+Y_{1}\right)\left[-1+2X_{1}\left(1+Y_{1}\right)\right]\sin\omega t\\ \dot{\eta_{2}}=0\;. \end{array}$$

The terms of the order of $\xi_{1}$, $\xi_{2}$, $\eta_{1}$, and $\eta_{2}$ have been neglected. Treating $X_{1}$, $X_{2}$, $Y_{1}$, and $Y_{2}$ as constants during the period of rapid oscillations 2π/ω and integrating Equation (A5) gives

(A6) $$\begin{array}{ccc} \xi_{1} & = & \frac{\epsilon }{\omega }X_{1}\left[-1-2Y_{1}+X_{1}\left(1+Y_{1}\right)\left(1+3Y_{1}\right)\right]\cos\omega t\\ \xi_{2} & = & 0\;\;(\mathrm{constant}\;\mathrm{of}\;\mathrm{integration}\;\mathrm{taken}\;\mathrm{as}\;\mathrm{zero})\\ \eta_{1} & = & -\frac{\epsilon }{\omega }Y_{1}\left(1+Y_{1}\right)\left[-1+2X_{1}\left(1+Y_{1}\right)\right]\cos\omega t\\ \eta_{2} & = & 0\;\;(\mathrm{constant}\;\mathrm{of}\;\mathrm{integration}\;\mathrm{taken}\;\mathrm{as}\;\mathrm{zero})\;. \end{array}$$

This transformation is canonical up to the second order of ${\left(\frac{\epsilon }{\omega }\right)}^{2}<<1$. The first non-vanishing correction to the Hamiltonian is of the order ${\left(\epsilon /\omega \right)}^{2}$ as well; thus, it is not sufficient. Therefore, we consider an almost-canonical transformation from the old variables $\left(q_{1},q_{2},p_{1},p_{2}\right)$ to the new variables $X_{1},X_{2},Y_{1},$ and $Y_{2}$:

(A7) $$\begin{array}{ccc} q_{1} & = & X_{1}+\frac{\epsilon \cos\omega t}{\omega }X_{1}\left[-1-2Y_{1}+X_{1}\left(1+Y_{1}\right)\left(1+3Y_{1}\right)\right]\\ & + & \frac{\epsilon^{2}\cos^{2}\omega t}{\omega^{2}}X_{1}\left[-1-2Y_{1}+X_{1}\left(1+Y_{1}\right)\left(1+3Y_{1}\right)\right]\left[-1-2Y_{1}+2X_{1}\left(1+Y_{1}\right)\left(1+3Y_{1}\right)\right]\\ q_{2} & = & X2\\ p_{1} & = & Y_{1}-\frac{\epsilon \cos\omega t}{\omega }Y_{1}\left(1+Y_{1}\right)\left[-1+2X_{1}\left(1+Y_{1}\right)\right]\\ & - & \frac{\epsilon^{2}\cos^{2}\omega t}{\omega^{2}}2X_{1}Y_{1}{\left(1+Y_{1}\right)}^{2}\left[-1-2Y_{1}+X_{1}\left(1+Y_{1}\right)\left(1+3Y_{1}\right)\right]\\ p_{2} & = & Y_{2} \end{array}$$

This transformation is canonical up to the third order of ${\left(\frac{\epsilon }{\omega }\right)}^{3}<<1$, as the Poisson brackets $\{q_{i},q_{j}\}=0$, $\{p_{i},p_{j}\}=0$, $\left\{q_{1},p_{1}\right\}=1+O{\left(\frac{\epsilon }{\omega }\right)}^{3}$, $\{q_{2},p_{2}\}=1$. The generating function of this transformation is given as

(A8) $$F_{2}=\frac{q_{1}Y_{1}\left(\omega -\left(1+Y_{1}\right)\left(-1+q_{1}+q_{1}Y_{1}\right)\epsilon \cos\omega t\right)}{\omega }\;.$$

The new Hamiltonian reads $H^{\prime }=H+\partial F_{2}/\partial t$. Averaging the new Hamiltonian $H^{\prime }$ over the period of rapid oscillations 2π/ω gives the following time-independent Hamiltonian:

(A9) $$\begin{array}{ccc} H^{\prime } & = & X_{1}Y_{1}\left(-B_{0}\left(Y_{1}+1\right)\left(X_{1}Y_{1}+X_{1}-1\right)-1\right)+\alpha X_{1}\left(Y_{2}-Y_{1}\right)+\beta X_{2}\left(Y_{1}-Y_{2}\right)\\ & + & \frac{X_{1}\epsilon^{2}}{2\omega^{2}}\left[\alpha Y_{2}-B_{0}X_{1}^{3}Y_{1}{\left(Y_{1}+1\right)}^{5}\left(3Y_{1}-1\right)+2X_{1}^{2}{\left(Y_{1}+1\right)}^{2}\right.\\ & & \left(2B_{0}Y_{1}^{4}+3Y_{1}^{3}\left(-\alpha +B_{0}-1\right)+Y_{1}\left(\alpha -B_{0}+6\alpha Y_{2}+1\right)+Y_{1}^{2}\left(2\alpha +9\alpha Y_{2}+2\right)+\alpha Y_{2}\right)\\ & - & X_{1}\left(Y_{1}+1\right)\left(3B_{0}Y_{1}^{4}+Y_{1}^{3}\left(-7\alpha +5B_{0}-7\right)+Y_{1}^{2}\left(\alpha +B_{0}+18\alpha Y_{2}+1\right)\right.\\ & + & \left.Y1\left(2\alpha -B_{0}+15\alpha Y_{2}+2\right)+3\alpha Y_{2}\right)+Y_{1}^{4}\left(B_{0}+4\beta X_{2}\right)\\ & + & 2Y_{1}^{3}\left(-\alpha +B_{0}+5\beta X_{2}-1\right)-2\beta X_{1}X_{2}{\left(Y_{1}+1\right)}^{3}\left(3Y_{1}+1\right)Y_{1}+2Y_{1}\left(\beta X_{2}+2\alpha Y_{2}\right)\\ & + & Y_{1}^{2}\left(-\alpha +B_{0}+8\beta X_{2}+4\alpha Y_{2}-1\right)\;. \end{array}$$

The resulting Hamilton’s equations are solved numerically to give the optimal path for which the action is calculated. In terms of the new coordinates and momenta, the fixed points become lengthy expressions, even more so for Hamilton’s equations.
